# 2-Amino-5-chloro­pyridinium 3-carb­oxy-4-hy­droxy­benzene­sulfonate

**DOI:** 10.1107/S1600536810032290

**Published:** 2010-08-18

**Authors:** Madhukar Hemamalini, Hoong-Kun Fun

**Affiliations:** a X-ray Crystallography Unit, School of Physics, Universiti Sains Malaysia, 11800 USM, Penang, Malaysia

## Abstract

The asymmetric unit of the title salt, C_5_H_6_ClN_2_^+^·C_7_H_5_O_6_S^−^, contains two independent 2-amino-5-chloro­pyridinium cations and two independent 3-carb­oxy-4-hy­droxy­benzene­sulfonate anions. In both anions, the O atoms of the sulfonate group are disordered over two sets of positions, with occupancy ratios of 0.47 (5):0.53 (5) and 0.50 (8):0.50 (8). In each anion, an intra­molecular O—H⋯O hydrogen bond generating an *S*(6) motif is observed. In the crystal structure, the cations and anions are linked *via* N—H⋯O, O—H⋯O and C—H⋯O hydrogen bonds, forming a two-dimensional network parallel to (110). The structure is further stabilized by π–π inter­actions between cations and anions [centroid–centroid distance = 3.5454 (12) Å]. The crystal studied was a non-merohedral twin, with a ratio of the twin components of 0.715 (3):0.285 (3).

## Related literature

For applications of inter­molecular inter­actions, see: Lam & Mak (2000 ▶). For sulfosalicylic acid complexes, see: Smith *et al.* (2004 ▶); Muthiah *et al.* (2003 ▶); Raj *et al.* (2003 ▶); Fan *et al.* (2005 ▶). For a related structure, see: Pourayoubi *et al.* (2007 ▶). For hydrogen-bond motifs, see: Bernstein *et al.* (1995 ▶). For bond-length data, see: Allen *et al.* (1987 ▶).
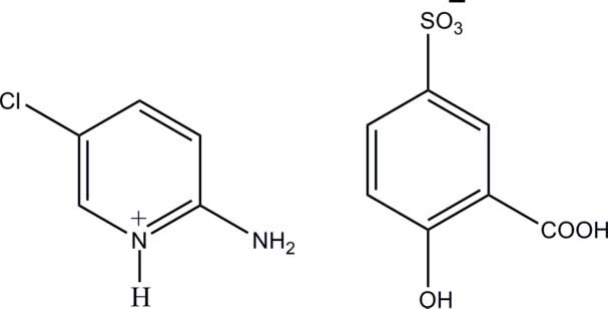


## Experimental

### 

#### Crystal data

C_5_H_6_ClN_2_^+^·C_7_H_5_O_6_S^−^
*M* *_r_* = 346.74
Triclinic, 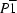

*a* = 7.9455 (3) Å
*b* = 10.9173 (5) Å
*c* = 16.3535 (7) Å
α = 85.223 (2)°
β = 83.327 (2)°
γ = 85.842 (2)°
*V* = 1401.22 (10) Å^3^
*Z* = 4
Mo *K*α radiation
μ = 0.45 mm^−1^
*T* = 296 K
0.50 × 0.36 × 0.15 mm

#### Data collection

Bruker SMART APEXII CCD area-detector diffractometer
Absorption correction: multi-scan (*SADABS*; Bruker, 2009 ▶) *T*_min_ = 0.804, *T*_max_ = 0.935
8143 measured reflections
8143 independent reflections
5951 reflections with *I* > 2σ(*I*)
*R*_int_ = 0.000

#### Refinement

*R*[*F* ^2^ > 2σ(*F* ^2^)] = 0.051
*wR*(*F* ^2^) = 0.158
*S* = 1.04
8143 reflections
478 parameters
6 restraints
H atoms treated by a mixture of independent and constrained refinement
Δρ_max_ = 0.54 e Å^−3^
Δρ_min_ = −0.42 e Å^−3^

### 

Data collection: *APEX2* (Bruker, 2009 ▶); cell refinement: *SAINT* (Bruker, 2009 ▶); data reduction: *SAINT*; program(s) used to solve structure: *SHELXTL* (Sheldrick, 2008 ▶); program(s) used to refine structure: *SHELXTL*; molecular graphics: *SHELXTL*; software used to prepare material for publication: *SHELXTL* and *PLATON* (Spek, 2009 ▶).

## Supplementary Material

Crystal structure: contains datablocks global, I. DOI: 10.1107/S1600536810032290/ci5152sup1.cif

Structure factors: contains datablocks I. DOI: 10.1107/S1600536810032290/ci5152Isup2.hkl

Additional supplementary materials:  crystallographic information; 3D view; checkCIF report

## Figures and Tables

**Table 1:** Hydrogen-bond geometry (Å, °)

*D*—H⋯*A*	*D*—H	H⋯*A*	*D*⋯*A*	*D*—H⋯*A*
N2*A*—H2*AA*⋯O6*X*^i^	0.86	2.08	2.900 (15)	159
N2*A*—H2*AB*⋯O5*X*^ii^	0.86	2.26	3.115 (9)	171
N2*B*—H2*BA*⋯O6*B*	0.86	2.34	3.114 (19)	150
N2*B*—H2*BA*⋯O6*Y*	0.86	2.20	2.985 (17)	152
N2*B*—H2*BB*⋯O5*B*^iii^	0.86	2.30	3.146 (17)	168
N2*B*—H2*BB*⋯O5*Y*^iii^	0.86	2.18	3.013 (15)	164
O3*A*—H2O*A*⋯O5*B*^iii^	0.83 (3)	1.83 (4)	2.657 (16)	180 (5)
O3*A*—H2O*A*⋯O5*Y*^iii^	0.83 (3)	1.84 (4)	2.663 (16)	173 (3)
O3*B*—H2O*B*⋯O5*X*^iv^	0.82 (3)	1.90 (3)	2.698 (10)	166 (3)
O1*B*—H1O*B*⋯O2*B*	0.83 (3)	1.84 (3)	2.604 (2)	152 (3)
O1*A*—H1O*A*⋯O2*A*	0.84 (3)	1.85 (3)	2.584 (2)	145 (3)
O1*A*—H1OO⋯O6*B*	0.84 (3)	2.51 (4)	3.086 (16)	127 (3)
O1*A*—H1O*A*⋯O6*Y*	0.84 (3)	2.58 (4)	3.163 (18)	128 (3)
N1*B*—H1N*B*⋯O4*B*	0.88 (3)	2.23 (4)	2.999 (19)	146 (3)
N1*B*—H1N*B*⋯O4*Y*	0.88 (3)	2.09 (3)	2.865 (13)	148 (3)
N1*A*—H1N*A*⋯O4*X*^i^	0.86 (3)	2.08 (4)	2.87 (2)	153 (3)
C1*A*—H1*AA*⋯O1*B*^v^	0.93	2.60	3.422 (3)	148

Symmetry codes: (i) 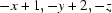; (ii) 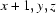; (iii) 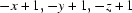; (iv) 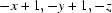; (v) 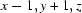.

**Fig. 1.**
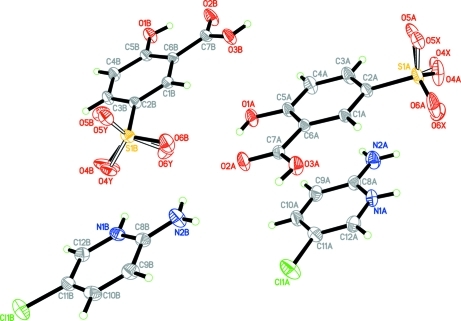
The asymmetric unit of the title compound. Displacement ellipsoids are drawn at the 30% probability level. Open bonds indicate minor disordered components.

**Fig. 2.**
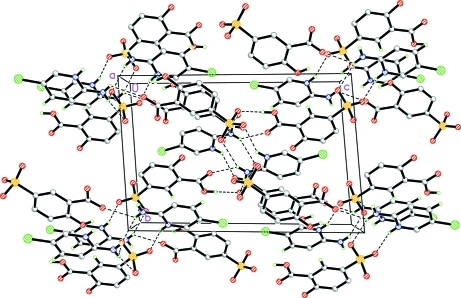
The crystal packing of the title compound, showing a hydrogen-bonded (dashed lines) network. H atoms not involved in the interactions have been omitted for clarity. Only the major component of the disordered O atoms of the sulfonate group are shown.

## supplementary crystallographic information

### Comment

Intermolecular interactions are responsible for crystal packing and gaining an understanding of them allows us to comprehend collective properties and permits the design of new crystals with specific physical and chemical properties (Lam & Mak, 2000). 5-Sulfosalicylic acid (3-carboxy-4-hydroxybenzenesulfonic acid) and its organic complexes or salts can develop well-defined non-covalent supramolecular architectures because of their ability to form multiple hydrogen bonds containing components of complementary arrays of hydrogen-bonding sites (Smith *et al.*, 2004; Muthiah *et al.*, 2003; Raj *et al.*, 2003; Fan *et al.*, 2005). The present study has been undertaken to study the hydrogen bonding patterns involving the 3-carboxy-4-hydroxybenzenesulfonate anions with the 2-amino-5-chloropyridinium cations.

The asymmetric unit of the title compound consists of two crystallographically independent 2-amino-5-chloropyridinium cations (A and B) and two 3-carboxy-4-hydroxybenzenesulfonate anions (A and B) (Fig. 1). Each 2-amino-5-chloropyridinium cation is planar, with a maximum deviation of 0.003 (2) Å for atom C8A in cation A and 0.013 (2) Å for C12B atom in cation B. In the cations, protonation at atoms N1A and N1B lead to a slight increase in the C8A—N1A—C12A [123.3 (2)°] and C8B—N1B—C12B [123.56 (19)°] angles compared to those observed in an unprotonated structure (Pourayoubi *et al.*, 2007). Bond lengths (Allen *et al.*, 1987) and angles are normal. Each 3-carboxy-4-hydroxybenzenesulfonate anions contains an intramolecular O—H···O hydrogen bond, which generates an *S*(6) ring.

In the crystal structure, (Fig. 2), the sulfonate group of each 3-carboxy-4-hydroxybenzenesulfonate anion interacts with the corresponding 2-amino-5-chloropyridinium cations via a pair of N—H···O hydrogen bonds forming an *R*_2_^2^(8) ring motif (Bernstein *et al.*, 1995). Here, sulfonate groups mimic the role of the carboxylate groups. The ionic units are further linked by N—H···O, O—H···O and C—H···O (Table 1) hydrogen bonds, forming a two dimensional network parallel to the (110) plane.

### Experimental

A hot methanol solution (20 ml) of 2-amino-5-chloropyridine (32 mg, Aldrich) and sulfosalicylic acid (54 mg, Merck) were mixed and warmed over a heating magnetic stirrer hotplate for a few minutes. The resulting solution was allowed to cool slowly at room temperature and crystals of the title compound appeared after a few days.

### Refinement

Atoms H2OA, H2OB, H1OA, H1OB, H1NA and H1NB were located in a difference Fourier map and refined freely. The remaining H atoms were positioned geometrically [C—H = 0.93 Å] and were refined using a riding model, with *U*_iso_(H) = 1.2*U*_eq_(C). The O atoms in the both sulfonate anions are disordered over two sets of positions, with occupancy ratios of 0.47 (5):0.53 (5) and 0.50 (8):0.50 (8). The crystal studied was a non-merohedral twin, the refined ratio of the two components being 0.715 (3):0.285 (3); the twin matrix is [-1 0 0.111, 0 -1 0.111, 0 0 1].

### Crystal data

**Table:**

C_5_H_6_ClN_2_^+^·C_7_H_5_O_6_S^−^	*Z* = 4
*M**_r_* = 346.74	*F*(000) = 712
Triclinic, *P*1	*D*_x_ = 1.644 Mg m^−^^3^
Hall symbol: -P 1	Mo *K*α radiation, λ = 0.71073 Å
*a* = 7.9455 (3) Å	Cell parameters from 9907 reflections
*b* = 10.9173 (5) Å	θ = 2.5–29.9°
*c* = 16.3535 (7) Å	µ = 0.45 mm^−^^1^
α = 85.223 (2)°	*T* = 296 K
β = 83.327 (2)°	Plate, yellow
γ = 85.842 (2)°	0.50 × 0.36 × 0.15 mm
*V* = 1401.22 (10) Å^3^	

### Data collection

**Table:**

Bruker SMART APEXII CCD area-detector diffractometer	8143 independent reflections
Radiation source: fine-focus sealed tube	5951 reflections with *I* > 2σ(*I*)
graphite	*R*_int_ = 0.000
φ and ω scans	θ_max_ = 30.1°, θ_min_ = 1.9°
Absorption correction: multi-scan (*SADABS*; Bruker, 2009)	*h* = −11→11
*T*_min_ = 0.804, *T*_max_ = 0.935	*k* = −15→15
8143 measured reflections	*l* = 0→22

### Refinement

**Table:**

Refinement on *F*^2^	Primary atom site location: structure-invariant direct methods
Least-squares matrix: full	Secondary atom site location: difference Fourier map
*R*[*F*^2^ > 2σ(*F*^2^)] = 0.051	Hydrogen site location: inferred from neighbouring sites
*wR*(*F*^2^) = 0.158	H atoms treated by a mixture of independent and constrained refinement
*S* = 1.04	*w* = 1/[σ^2^(*F*_o_^2^) + (0.0866*P*)^2^ + 0.2919*P*] where *P* = (*F*_o_^2^ + 2*F*_c_^2^)/3
8143 reflections	(Δ/σ)_max_ = 0.001
478 parameters	Δρ_max_ = 0.54 e Å^−^^3^
6 restraints	Δρ_min_ = −0.42 e Å^−^^3^

### Special details

**Table:**

Geometry. All esds (except the esd in the dihedral angle between two l.s. planes) are estimated using the full covariance matrix. The cell esds are taken into account individually in the estimation of esds in distances, angles and torsion angles; correlations between esds in cell parameters are only used when they are defined by crystal symmetry. An approximate (isotropic) treatment of cell esds is used for estimating esds involving l.s. planes.
Refinement. Refinement of F^2^ against ALL reflections. The weighted R-factor wR and goodness of fit S are based on F^2^, conventional R-factors R are based on F, with F set to zero for negative F^2^. The threshold expression of F^2^ > 2sigma(F^2^) is used only for calculating R-factors(gt) etc. and is not relevant to the choice of reflections for refinement. R-factors based on F^2^ are statistically about twice as large as those based on F, and R- factors based on ALL data will be even larger.

### Fractional atomic coordinates and isotropic or equivalent isotropic displacement parameters (Å^2^)

**Table:**

	*x*	*y*	*z*	*U*_iso_*/*U*_eq_	Occ. (<1)
S1A	0.35527 (7)	0.80743 (5)	0.00751 (3)	0.04328 (15)	
O1A	0.7640 (2)	0.53220 (16)	0.24854 (11)	0.0476 (4)	
O2A	0.5653 (2)	0.63703 (16)	0.36240 (9)	0.0513 (4)	
O3A	0.3497 (2)	0.76513 (15)	0.32837 (10)	0.0455 (4)	
O4A	0.460 (2)	0.837 (2)	−0.0643 (13)	0.072 (5)	0.47 (5)
O5A	0.233 (3)	0.711 (3)	−0.0064 (12)	0.086 (4)	0.47 (5)
O6A	0.266 (3)	0.906 (2)	0.0472 (9)	0.075 (4)	0.47 (5)
O4X	0.465 (2)	0.822 (2)	−0.0720 (11)	0.061 (3)	0.53 (5)
O5X	0.2139 (10)	0.7391 (10)	0.0023 (6)	0.045 (2)	0.53 (5)
O6X	0.304 (2)	0.9285 (11)	0.0377 (11)	0.074 (3)	0.53 (5)
C1A	0.4393 (2)	0.74515 (17)	0.16239 (11)	0.0312 (4)	
H1AA	0.3498	0.8004	0.1792	0.037*	
C2A	0.4802 (3)	0.72879 (19)	0.07962 (12)	0.0352 (4)	
C3A	0.6153 (3)	0.6469 (2)	0.05434 (14)	0.0496 (6)	
H3AA	0.6427	0.6358	−0.0015	0.060*	
C4A	0.7087 (3)	0.5821 (2)	0.11146 (14)	0.0506 (6)	
H4AA	0.7989	0.5279	0.0940	0.061*	
C5A	0.6684 (2)	0.59768 (18)	0.19527 (12)	0.0352 (4)	
C6A	0.5314 (2)	0.67910 (16)	0.22106 (11)	0.0291 (4)	
C7A	0.4856 (2)	0.69252 (17)	0.30980 (12)	0.0327 (4)	
Cl1A	0.65336 (10)	0.96478 (9)	0.40572 (4)	0.0741 (2)	
N1A	0.6964 (2)	0.97630 (18)	0.16358 (12)	0.0434 (4)	
N2A	0.8778 (3)	0.8864 (2)	0.06209 (13)	0.0584 (6)	
H2AA	0.8203	0.9251	0.0255	0.070*	
H2AB	0.9646	0.8383	0.0472	0.070*	
C8A	0.8330 (3)	0.9006 (2)	0.14072 (14)	0.0415 (5)	
C9A	0.9200 (3)	0.8392 (2)	0.20505 (16)	0.0469 (5)	
H9AA	1.0138	0.7854	0.1922	0.056*	
C10A	0.8675 (3)	0.8583 (2)	0.28486 (15)	0.0460 (5)	
H10A	0.9259	0.8186	0.3266	0.055*	
C11A	0.7244 (3)	0.9383 (2)	0.30458 (14)	0.0438 (5)	
C12A	0.6419 (3)	0.9960 (2)	0.24326 (15)	0.0448 (5)	
H12A	0.5475	1.0494	0.2557	0.054*	
S1B	0.85425 (6)	0.30940 (5)	0.47122 (3)	0.03661 (13)	
O1B	1.2477 (2)	0.02225 (16)	0.21626 (10)	0.0469 (4)	
O2B	1.0653 (2)	0.14325 (18)	0.11015 (10)	0.0600 (5)	
O3B	0.8506 (2)	0.27142 (16)	0.15525 (11)	0.0530 (4)	
O4B	0.9689 (18)	0.322 (2)	0.5284 (9)	0.078 (5)	0.50 (8)
O5B	0.7188 (19)	0.226 (2)	0.5092 (10)	0.053 (3)	0.50 (8)
O6B	0.784 (2)	0.4156 (19)	0.4259 (9)	0.061 (3)	0.50 (8)
O4Y	0.9709 (15)	0.3365 (13)	0.5308 (7)	0.042 (3)	0.50 (8)
O5Y	0.7124 (15)	0.2449 (19)	0.5077 (10)	0.041 (2)	0.50 (8)
O6Y	0.802 (2)	0.4296 (15)	0.4326 (11)	0.059 (3)	0.50 (8)
C1B	0.9347 (2)	0.24440 (17)	0.31394 (12)	0.0309 (4)	
H1BA	0.8522	0.3052	0.3003	0.037*	
C2B	0.9681 (2)	0.22231 (17)	0.39468 (11)	0.0310 (4)	
C3B	1.0904 (3)	0.13016 (19)	0.41552 (12)	0.0370 (4)	
H3BA	1.1114	0.1140	0.4703	0.044*	
C4B	1.1802 (3)	0.06305 (19)	0.35518 (13)	0.0390 (4)	
H4BA	1.2607	0.0013	0.3695	0.047*	
C5B	1.1508 (2)	0.08727 (17)	0.27276 (12)	0.0323 (4)	
C6B	1.0231 (2)	0.17659 (16)	0.25225 (11)	0.0292 (4)	
C7B	0.9840 (3)	0.19622 (19)	0.16594 (13)	0.0371 (4)	
Cl1B	0.81241 (10)	0.51032 (8)	0.86096 (4)	0.0679 (2)	
N1B	0.7949 (2)	0.52963 (17)	0.62141 (11)	0.0374 (4)	
N2B	0.6228 (3)	0.6290 (2)	0.53013 (13)	0.0544 (5)	
H2BA	0.6839	0.5943	0.4905	0.065*	
H2BB	0.5371	0.6782	0.5199	0.065*	
C8B	0.6606 (3)	0.60676 (19)	0.60619 (13)	0.0375 (4)	
C9B	0.5673 (3)	0.6602 (2)	0.67497 (15)	0.0450 (5)	
H9BA	0.4743	0.7149	0.6671	0.054*	
C10B	0.6122 (3)	0.6323 (2)	0.75211 (14)	0.0456 (5)	
H10B	0.5497	0.6669	0.7971	0.055*	
C11B	0.7538 (3)	0.5506 (2)	0.76350 (13)	0.0402 (4)	
C12B	0.8428 (3)	0.5010 (2)	0.69775 (14)	0.0397 (4)	
H12B	0.9370	0.4471	0.7048	0.048*	
H2OA	0.328 (4)	0.768 (3)	0.379 (2)	0.075 (10)*	
H2OB	0.840 (4)	0.279 (3)	0.106 (2)	0.062 (9)*	
H1OB	1.210 (4)	0.046 (3)	0.172 (2)	0.058 (8)*	
H1OA	0.724 (4)	0.545 (3)	0.297 (2)	0.069 (9)*	
H1NB	0.855 (3)	0.496 (3)	0.5796 (19)	0.055 (8)*	
H1NA	0.644 (4)	1.017 (3)	0.126 (2)	0.065 (9)*	

### Atomic displacement parameters (Å^2^)

**Table:**

	*U*^11^	*U*^22^	*U*^33^	*U*^12^	*U*^13^	*U*^23^
S1A	0.0503 (3)	0.0502 (3)	0.0293 (3)	0.0092 (2)	−0.0148 (2)	0.0015 (2)
O1A	0.0462 (9)	0.0562 (10)	0.0372 (8)	0.0221 (7)	−0.0100 (7)	0.0004 (7)
O2A	0.0643 (10)	0.0587 (10)	0.0276 (7)	0.0213 (8)	−0.0099 (7)	0.0010 (7)
O3A	0.0515 (9)	0.0503 (9)	0.0298 (7)	0.0173 (7)	0.0029 (6)	−0.0019 (6)
O4A	0.070 (7)	0.085 (6)	0.049 (8)	0.030 (5)	0.002 (5)	0.027 (5)
O5A	0.125 (9)	0.081 (8)	0.066 (6)	−0.031 (6)	−0.071 (6)	0.024 (5)
O6A	0.088 (7)	0.093 (9)	0.037 (3)	0.063 (6)	−0.017 (4)	−0.007 (5)
O4X	0.061 (6)	0.093 (7)	0.027 (3)	−0.001 (5)	−0.007 (3)	0.011 (4)
O5X	0.044 (4)	0.055 (3)	0.036 (2)	0.002 (2)	−0.015 (3)	0.002 (2)
O6X	0.107 (7)	0.039 (3)	0.078 (7)	0.019 (4)	−0.040 (5)	−0.007 (3)
C1A	0.0317 (9)	0.0326 (9)	0.0295 (9)	0.0047 (7)	−0.0072 (7)	−0.0037 (7)
C2A	0.0392 (10)	0.0390 (10)	0.0280 (9)	0.0057 (8)	−0.0099 (7)	−0.0036 (7)
C3A	0.0586 (14)	0.0608 (14)	0.0274 (10)	0.0219 (11)	−0.0074 (9)	−0.0101 (9)
C4A	0.0527 (13)	0.0596 (14)	0.0357 (11)	0.0279 (11)	−0.0050 (9)	−0.0090 (10)
C5A	0.0363 (9)	0.0375 (10)	0.0311 (9)	0.0089 (8)	−0.0075 (7)	−0.0023 (8)
C6A	0.0302 (8)	0.0300 (9)	0.0270 (8)	0.0029 (7)	−0.0046 (6)	−0.0032 (7)
C7A	0.0385 (9)	0.0305 (9)	0.0282 (9)	0.0017 (7)	−0.0036 (7)	−0.0010 (7)
Cl1A	0.0750 (5)	0.1075 (6)	0.0413 (3)	0.0024 (4)	−0.0094 (3)	−0.0165 (4)
N1A	0.0434 (10)	0.0474 (10)	0.0375 (9)	0.0002 (8)	−0.0086 (8)	0.0109 (8)
N2A	0.0645 (13)	0.0678 (14)	0.0390 (11)	0.0034 (11)	0.0013 (9)	0.0032 (10)
C8A	0.0443 (11)	0.0414 (11)	0.0380 (11)	−0.0072 (9)	−0.0035 (9)	0.0042 (9)
C9A	0.0401 (11)	0.0455 (12)	0.0539 (13)	0.0031 (9)	−0.0080 (10)	0.0021 (10)
C10A	0.0472 (12)	0.0487 (12)	0.0435 (12)	−0.0025 (10)	−0.0173 (9)	0.0056 (10)
C11A	0.0428 (11)	0.0520 (13)	0.0375 (11)	−0.0071 (10)	−0.0071 (9)	−0.0022 (9)
C12A	0.0405 (11)	0.0436 (12)	0.0485 (12)	0.0016 (9)	−0.0036 (9)	0.0007 (10)
S1B	0.0375 (3)	0.0410 (3)	0.0302 (2)	0.00613 (19)	0.00170 (18)	−0.01102 (19)
O1B	0.0503 (9)	0.0549 (10)	0.0337 (8)	0.0255 (7)	−0.0069 (7)	−0.0143 (7)
O2B	0.0735 (12)	0.0750 (12)	0.0305 (8)	0.0284 (10)	−0.0126 (8)	−0.0174 (8)
O3B	0.0641 (11)	0.0569 (10)	0.0401 (9)	0.0240 (8)	−0.0265 (8)	−0.0124 (8)
O4B	0.051 (5)	0.115 (10)	0.076 (7)	0.002 (5)	−0.006 (4)	−0.067 (7)
O5B	0.067 (7)	0.052 (5)	0.039 (4)	−0.015 (4)	0.022 (4)	−0.018 (3)
O6B	0.079 (5)	0.057 (6)	0.043 (3)	0.041 (5)	−0.012 (5)	−0.014 (3)
O4Y	0.044 (4)	0.049 (5)	0.035 (4)	0.006 (2)	−0.009 (3)	−0.024 (3)
O5Y	0.035 (4)	0.053 (5)	0.035 (4)	0.004 (3)	−0.001 (3)	−0.007 (3)
O6Y	0.078 (5)	0.033 (3)	0.058 (6)	0.007 (4)	0.028 (5)	−0.009 (3)
C1B	0.0306 (8)	0.0294 (9)	0.0324 (9)	0.0055 (7)	−0.0051 (7)	−0.0053 (7)
C2B	0.0299 (8)	0.0334 (9)	0.0292 (9)	0.0036 (7)	−0.0006 (7)	−0.0077 (7)
C3B	0.0416 (10)	0.0429 (11)	0.0259 (9)	0.0084 (8)	−0.0077 (7)	−0.0027 (8)
C4B	0.0421 (10)	0.0386 (10)	0.0349 (10)	0.0146 (8)	−0.0082 (8)	−0.0044 (8)
C5B	0.0329 (9)	0.0323 (9)	0.0316 (9)	0.0073 (7)	−0.0044 (7)	−0.0092 (7)
C6B	0.0308 (8)	0.0293 (8)	0.0284 (8)	0.0027 (7)	−0.0069 (7)	−0.0059 (7)
C7B	0.0446 (11)	0.0368 (10)	0.0314 (9)	0.0057 (8)	−0.0117 (8)	−0.0077 (8)
Cl1B	0.0757 (5)	0.0929 (5)	0.0356 (3)	−0.0065 (4)	−0.0114 (3)	0.0010 (3)
N1B	0.0349 (8)	0.0443 (10)	0.0331 (8)	0.0013 (7)	0.0007 (7)	−0.0125 (7)
N2B	0.0505 (11)	0.0758 (15)	0.0366 (10)	0.0078 (10)	−0.0084 (8)	−0.0080 (10)
C8B	0.0354 (10)	0.0415 (11)	0.0357 (10)	−0.0041 (8)	−0.0011 (8)	−0.0070 (8)
C9B	0.0405 (11)	0.0451 (12)	0.0480 (12)	0.0086 (9)	−0.0007 (9)	−0.0113 (10)
C10B	0.0476 (12)	0.0508 (13)	0.0379 (11)	−0.0012 (10)	0.0055 (9)	−0.0166 (9)
C11B	0.0465 (11)	0.0437 (11)	0.0315 (10)	−0.0092 (9)	−0.0047 (8)	−0.0040 (8)
C12B	0.0377 (10)	0.0396 (11)	0.0421 (11)	−0.0010 (8)	−0.0048 (8)	−0.0052 (9)

### Geometric parameters (Å, °)

**Table:**

S1A—O4A	1.386 (18)	S1B—O4B	1.400 (15)
S1A—O5X	1.407 (10)	S1B—O5Y	1.418 (14)
S1A—O6A	1.411 (13)	S1B—O6B	1.436 (11)
S1A—O6X	1.463 (12)	S1B—O6Y	1.462 (12)
S1A—O4X	1.481 (16)	S1B—O4Y	1.481 (13)
S1A—O5A	1.527 (17)	S1B—O5B	1.502 (17)
S1A—C2A	1.7586 (19)	S1B—C2B	1.7605 (18)
O1A—C5A	1.348 (2)	O1B—C5B	1.347 (2)
O1A—H1OA	0.84 (3)	O1B—H1OB	0.84 (3)
O2A—C7A	1.222 (2)	O2B—C7B	1.217 (3)
O3A—C7A	1.314 (2)	O3B—C7B	1.312 (2)
O3A—H2OA	0.83 (4)	O3B—H2OB	0.82 (3)
C1A—C2A	1.378 (3)	C1B—C2B	1.375 (3)
C1A—C6A	1.396 (2)	C1B—C6B	1.394 (2)
C1A—H1AA	0.93	C1B—H1BA	0.93
C2A—C3A	1.394 (3)	C2B—C3B	1.397 (3)
C3A—C4A	1.378 (3)	C3B—C4B	1.378 (3)
C3A—H3AA	0.93	C3B—H3BA	0.93
C4A—C5A	1.392 (3)	C4B—C5B	1.395 (3)
C4A—H4AA	0.93	C4B—H4BA	0.93
C5A—C6A	1.403 (2)	C5B—C6B	1.405 (2)
C6A—C7A	1.472 (3)	C6B—C7B	1.475 (3)
Cl1A—C11A	1.725 (2)	Cl1B—C11B	1.725 (2)
N1A—C8A	1.352 (3)	N1B—C8B	1.344 (3)
N1A—C12A	1.354 (3)	N1B—C12B	1.353 (3)
N1A—H1NA	0.85 (3)	N1B—H1NB	0.88 (3)
N2A—C8A	1.313 (3)	N2B—C8B	1.312 (3)
N2A—H2AA	0.86	N2B—H2BA	0.86
N2A—H2AB	0.86	N2B—H2BB	0.86
C8A—C9A	1.421 (3)	C8B—C9B	1.417 (3)
C9A—C10A	1.352 (4)	C9B—C10B	1.355 (3)
C9A—H9AA	0.93	C9B—H9BA	0.93
C10A—C11A	1.406 (3)	C10B—C11B	1.404 (3)
C10A—H10A	0.93	C10B—H10B	0.93
C11A—C12A	1.350 (3)	C11B—C12B	1.347 (3)
C12A—H12A	0.93	C12B—H12B	0.93
			
O4A—S1A—O5X	118.5 (11)	O4B—S1B—O5Y	111.5 (9)
O4A—S1A—O6A	116.7 (12)	O4B—S1B—O6B	120.8 (18)
O5X—S1A—O6A	96.8 (11)	O5Y—S1B—O6B	105.3 (9)
O4A—S1A—O6X	102.2 (12)	O4B—S1B—O6Y	110.8 (18)
O5X—S1A—O6X	111.5 (7)	O5Y—S1B—O6Y	111.5 (7)
O5X—S1A—O4X	112.8 (8)	O5Y—S1B—O4Y	113.8 (7)
O6A—S1A—O4X	123.8 (12)	O6B—S1B—O4Y	115.0 (14)
O6X—S1A—O4X	109.6 (9)	O6Y—S1B—O4Y	105.0 (13)
O4A—S1A—O5A	111.0 (11)	O4B—S1B—O5B	109.7 (10)
O6A—S1A—O5A	110.2 (8)	O6B—S1B—O5B	111.7 (7)
O6X—S1A—O5A	124.9 (10)	O6Y—S1B—O5B	118.3 (9)
O4X—S1A—O5A	104.3 (10)	O4Y—S1B—O5B	112.6 (8)
O4A—S1A—C2A	108.6 (9)	O4B—S1B—C2B	105.3 (8)
O5X—S1A—C2A	108.6 (5)	O5Y—S1B—C2B	109.0 (8)
O6A—S1A—C2A	106.6 (6)	O6B—S1B—C2B	104.2 (7)
O6X—S1A—C2A	106.7 (6)	O6Y—S1B—C2B	108.4 (7)
O4X—S1A—C2A	107.3 (8)	O4Y—S1B—C2B	109.0 (5)
O5A—S1A—C2A	102.7 (7)	O5B—S1B—C2B	103.3 (9)
C5A—O1A—H1OA	110 (2)	C5B—O1B—H1OB	104.2 (19)
C7A—O3A—H2OA	111 (2)	C7B—O3B—H2OB	108 (2)
C2A—C1A—C6A	120.28 (17)	C2B—C1B—C6B	120.67 (16)
C2A—C1A—H1AA	119.9	C2B—C1B—H1BA	119.7
C6A—C1A—H1AA	119.9	C6B—C1B—H1BA	119.7
C1A—C2A—C3A	119.85 (18)	C1B—C2B—C3B	119.86 (17)
C1A—C2A—S1A	119.28 (15)	C1B—C2B—S1B	119.63 (14)
C3A—C2A—S1A	120.82 (15)	C3B—C2B—S1B	120.51 (15)
C4A—C3A—C2A	120.5 (2)	C4B—C3B—C2B	120.22 (18)
C4A—C3A—H3AA	119.7	C4B—C3B—H3BA	119.9
C2A—C3A—H3AA	119.7	C2B—C3B—H3BA	119.9
C3A—C4A—C5A	120.18 (19)	C3B—C4B—C5B	120.35 (17)
C3A—C4A—H4AA	119.9	C3B—C4B—H4BA	119.8
C5A—C4A—H4AA	119.9	C5B—C4B—H4BA	119.8
O1A—C5A—C4A	117.78 (18)	O1B—C5B—C4B	117.54 (17)
O1A—C5A—C6A	122.72 (18)	O1B—C5B—C6B	123.07 (17)
C4A—C5A—C6A	119.49 (18)	C4B—C5B—C6B	119.39 (17)
C1A—C6A—C5A	119.67 (17)	C1B—C6B—C5B	119.41 (17)
C1A—C6A—C7A	120.87 (16)	C1B—C6B—C7B	121.17 (16)
C5A—C6A—C7A	119.46 (16)	C5B—C6B—C7B	119.41 (17)
O2A—C7A—O3A	122.55 (18)	O2B—C7B—O3B	123.09 (19)
O2A—C7A—C6A	122.24 (18)	O2B—C7B—C6B	122.51 (18)
O3A—C7A—C6A	115.18 (17)	O3B—C7B—C6B	114.34 (18)
C8A—N1A—C12A	123.3 (2)	C8B—N1B—C12B	123.56 (19)
C8A—N1A—H1NA	119 (2)	C8B—N1B—H1NB	118.3 (19)
C12A—N1A—H1NA	117 (2)	C12B—N1B—H1NB	118.1 (19)
C8A—N2A—H2AA	120.0	C8B—N2B—H2BA	120.0
C8A—N2A—H2AB	120.0	C8B—N2B—H2BB	120.0
H2AA—N2A—H2AB	120.0	H2BA—N2B—H2BB	120.0
N2A—C8A—N1A	119.6 (2)	N2B—C8B—N1B	119.5 (2)
N2A—C8A—C9A	123.5 (2)	N2B—C8B—C9B	123.7 (2)
N1A—C8A—C9A	116.9 (2)	N1B—C8B—C9B	116.86 (19)
C10A—C9A—C8A	120.5 (2)	C10B—C9B—C8B	120.7 (2)
C10A—C9A—H9AA	119.8	C10B—C9B—H9BA	119.7
C8A—C9A—H9AA	119.8	C8B—C9B—H9BA	119.7
C9A—C10A—C11A	119.9 (2)	C9B—C10B—C11B	119.4 (2)
C9A—C10A—H10A	120.1	C9B—C10B—H10B	120.3
C11A—C10A—H10A	120.1	C11B—C10B—H10B	120.3
C12A—C11A—C10A	119.4 (2)	C12B—C11B—C10B	119.6 (2)
C12A—C11A—Cl1A	119.39 (19)	C12B—C11B—Cl1B	119.53 (18)
C10A—C11A—Cl1A	121.21 (18)	C10B—C11B—Cl1B	120.81 (17)
C11A—C12A—N1A	120.1 (2)	C11B—C12B—N1B	119.9 (2)
C11A—C12A—H12A	120.0	C11B—C12B—H12B	120.1
N1A—C12A—H12A	120.0	N1B—C12B—H12B	120.1
			
C6A—C1A—C2A—C3A	−0.7 (3)	C6B—C1B—C2B—C3B	−0.7 (3)
C6A—C1A—C2A—S1A	176.84 (15)	C6B—C1B—C2B—S1B	−179.98 (14)
O4A—S1A—C2A—C1A	145.7 (11)	O4B—S1B—C2B—C1B	−146.3 (9)
O5X—S1A—C2A—C1A	−84.1 (4)	O5Y—S1B—C2B—C1B	93.8 (6)
O6A—S1A—C2A—C1A	19.2 (13)	O6B—S1B—C2B—C1B	−18.2 (11)
O6X—S1A—C2A—C1A	36.2 (8)	O6Y—S1B—C2B—C1B	−27.7 (10)
O4X—S1A—C2A—C1A	153.7 (9)	O4Y—S1B—C2B—C1B	−141.4 (5)
O5A—S1A—C2A—C1A	−96.7 (12)	O5B—S1B—C2B—C1B	98.6 (7)
O4A—S1A—C2A—C3A	−36.8 (11)	O4B—S1B—C2B—C3B	34.4 (9)
O5X—S1A—C2A—C3A	93.4 (4)	O5Y—S1B—C2B—C3B	−85.4 (6)
O6A—S1A—C2A—C3A	−163.3 (13)	O6B—S1B—C2B—C3B	162.5 (11)
O6X—S1A—C2A—C3A	−146.3 (8)	O6Y—S1B—C2B—C3B	153.1 (10)
O4X—S1A—C2A—C3A	−28.8 (9)	O4Y—S1B—C2B—C3B	39.3 (5)
O5A—S1A—C2A—C3A	80.8 (12)	O5B—S1B—C2B—C3B	−80.6 (8)
C1A—C2A—C3A—C4A	−0.1 (4)	C1B—C2B—C3B—C4B	1.3 (3)
S1A—C2A—C3A—C4A	−177.6 (2)	S1B—C2B—C3B—C4B	−179.42 (17)
C2A—C3A—C4A—C5A	0.2 (4)	C2B—C3B—C4B—C5B	0.6 (3)
C3A—C4A—C5A—O1A	−179.9 (2)	C3B—C4B—C5B—O1B	177.2 (2)
C3A—C4A—C5A—C6A	0.4 (4)	C3B—C4B—C5B—C6B	−3.1 (3)
C2A—C1A—C6A—C5A	1.3 (3)	C2B—C1B—C6B—C5B	−1.8 (3)
C2A—C1A—C6A—C7A	−177.93 (18)	C2B—C1B—C6B—C7B	177.69 (18)
O1A—C5A—C6A—C1A	179.19 (19)	O1B—C5B—C6B—C1B	−176.61 (18)
C4A—C5A—C6A—C1A	−1.2 (3)	C4B—C5B—C6B—C1B	3.7 (3)
O1A—C5A—C6A—C7A	−1.6 (3)	O1B—C5B—C6B—C7B	3.9 (3)
C4A—C5A—C6A—C7A	178.1 (2)	C4B—C5B—C6B—C7B	−175.82 (19)
C1A—C6A—C7A—O2A	−179.1 (2)	C1B—C6B—C7B—O2B	175.9 (2)
C5A—C6A—C7A—O2A	1.7 (3)	C5B—C6B—C7B—O2B	−4.7 (3)
C1A—C6A—C7A—O3A	3.1 (3)	C1B—C6B—C7B—O3B	−6.8 (3)
C5A—C6A—C7A—O3A	−176.13 (18)	C5B—C6B—C7B—O3B	172.69 (18)
C12A—N1A—C8A—N2A	−180.0 (2)	C12B—N1B—C8B—N2B	−179.7 (2)
C12A—N1A—C8A—C9A	0.6 (3)	C12B—N1B—C8B—C9B	0.5 (3)
N2A—C8A—C9A—C10A	179.8 (2)	N2B—C8B—C9B—C10B	179.4 (2)
N1A—C8A—C9A—C10A	−0.8 (3)	N1B—C8B—C9B—C10B	−0.8 (3)
C8A—C9A—C10A—C11A	0.8 (4)	C8B—C9B—C10B—C11B	0.7 (4)
C9A—C10A—C11A—C12A	−0.6 (3)	C9B—C10B—C11B—C12B	−0.1 (3)
C9A—C10A—C11A—Cl1A	179.90 (19)	C9B—C10B—C11B—Cl1B	−178.64 (18)
C10A—C11A—C12A—N1A	0.3 (3)	C10B—C11B—C12B—N1B	−0.3 (3)
Cl1A—C11A—C12A—N1A	179.87 (17)	Cl1B—C11B—C12B—N1B	178.26 (16)
C8A—N1A—C12A—C11A	−0.4 (4)	C8B—N1B—C12B—C11B	0.1 (3)

### Hydrogen-bond geometry (Å, °)

**Table:**

*D*—H···*A*	*D*—H	H···*A*	*D*···*A*	*D*—H···*A*
N2A—H2AA···O6X^i^	0.86	2.08	2.900 (15)	159
N2A—H2AB···O5X^ii^	0.86	2.26	3.115 (9)	171
N2B—H2BA···O6B	0.86	2.34	3.114 (19)	150
N2B—H2BA···O6Y	0.86	2.20	2.985 (17)	152
N2B—H2BB···O5B^iii^	0.86	2.30	3.146 (17)	168
N2B—H2BB···O5Y^iii^	0.86	2.18	3.013 (15)	164
O3A—H2OA···O5B^iii^	0.83 (3)	1.83 (4)	2.657 (16)	180 (5)
O3A—H2OA···O5Y^iii^	0.83 (3)	1.84 (4)	2.663 (16)	173 (3)
O3B—H2OB···O5X^iv^	0.82 (3)	1.90 (3)	2.698 (10)	166 (3)
O1B—H1OB···O2B	0.83 (3)	1.84 (3)	2.604 (2)	152 (3)
O1A—H1OA···O2A	0.84 (3)	1.85 (3)	2.584 (2)	145 (3)
O1A—H1OA···O6B	0.84 (3)	2.51 (4)	3.086 (16)	127 (3)
O1A—H1OA···O6Y	0.84 (3)	2.58 (4)	3.163 (18)	128 (3)
N1B—H1NB···O4B	0.88 (3)	2.23 (4)	2.999 (19)	146 (3)
N1B—H1NB···O4Y	0.88 (3)	2.09 (3)	2.865 (13)	148 (3)
N1A—H1NA···O4X^i^	0.86 (3)	2.08 (4)	2.87 (2)	153 (3)
C1A—H1AA···O1B^v^	0.93	2.60	3.422 (3)	148

Symmetry codes: (i) −*x*+1, −*y*+2, −*z*; (ii) *x*+1, *y*, *z*; (iii) −*x*+1, −*y*+1, −*z*+1; (iv) −*x*+1, −*y*+1, −*z*; (v) *x*−1, *y*+1, *z*.
